# Current Concepts in the Management of Sanfilippo Syndrome (MPS III): A Narrative Review

**DOI:** 10.7759/cureus.58023

**Published:** 2024-04-11

**Authors:** Anas S Alyazidi, Osama Y Muthaffar, Layan S Baaishrah, Mohammed K Shawli, Abdulaziz T Jambi, Maram A Aljezani, Majdah A Almaghrabi

**Affiliations:** 1 Pediatrics, King Abdulaziz University Faculty of Medicine, Jeddah, SAU; 2 Faculty of Pharmacy, King Abdulaziz University Hospital, Jeddah, SAU; 3 Medicine, King Abdulaziz University Faculty of Medicine, Jeddah, SAU; 4 Pediatric Neurology, King Abdulaziz University Hospital, Jeddah, SAU

**Keywords:** nervous system, glycosaminoglycans, enzyme replacement therapy, gene therapy, stem cell, mucopolysaccharidosis iii, sanfilippo syndrome

## Abstract

Sanfilippo syndrome is a childhood-onset (1-4 years) autosomal recessive lysosomal storage disease that presents as a neurodegenerative disease by targeting the brain and spinal cord. It is also known as mucopolysaccharidosis III. Mucopolysaccharidosis III is divided into four subtypes (A, B, C, or D). It can cause delayed speech, behavior problems, and features of autism spectrum disorder. Sanfilippo syndrome is of a higher prevalence within consanguineous families that carry its gene alteration. If both parents have a nonfunctional copy of a gene linked to this condition, their children will have a 25% (1 in 4) chance of developing the disease. In Saudi Arabia, the incidence rate is estimated at 2 per 100,000 live births. Recent research focused on promising treatment approaches, such as gene therapy, modified enzyme replacement therapy, and stem cells. These approaches work by exogenous administration of the proper version of the mutant enzyme (enzyme replacement therapy), cleaning the defective enzyme in individuals with glycolipid storage disorders (substrate reduction therapy), or using a pharmacological chaperone to target improperly folded proteins. However, there is currently no approved curative medication for Sanfilippo syndrome that can effectively halt or reverse the disorder.

## Introduction and background

Sanfilippo syndrome, also known as mucopolysaccharidosis type III (MPS III), is a type of neurodegenerative lysosomal storage disease inherited as autosomal recessive that mainly affects the brain and spinal cord (nervous system), leading to a neurocognitive degeneration. A deficiency or mutation in the lysosome that breaks down glycosaminoglycans (GAGs) or mucopolysaccharides (MPSs) may result in the development of Sanfilippo syndrome. GAGs aid the body in the formation of cartilage, connective tissue, nerve tissue, skin, and blood clots, as well as in cell communication. Sanfilippo syndrome is characterized by cognitive deterioration, hyperactivity, intellectual decline, speech delay and progressive motor deterioration [1]. Lysosomal storage disorders are a heterogenous group of at least 41 genetically different but biochemically linked diseases. In general, neurodegenerative disorders have a negative impact on affected individuals and their families, and they consume a large amount of healthcare resources [2]. Compared to mucopolysaccharidosis IH and II, mucopolysaccharidosis III is considered the most common of these genetic disorders (Table 1).

**Table 1:** Distribution of total mutations described for each Sanfilippo syndrome (MPS III) subtype.

Subtype (Gene)	Total Mutations	Missense/Nonsense	Small Deletions	Small Insertions	Small Indels	Splicing	Gross Deletions	Gross Insertions and Duplications	Complex Rearrangements
MPS III A (SGSH)	155	118	20	9	1	3	3	1	0
MPS III B (NAGLU)	229	167	29	16	1	8	4	4	0
MPS III C (HGSNAT)	77	43	6	6	1	15	4	1	1
MPS III D (GNS)	25	7	5	4	1	4	2	0	2

In this review, we will primarily focus on highlighting mucopolysaccharidosis III treatment options and intervention techniques. Mucopolysaccharidosis III is a rare genetic disease. It is one of the causes of infantile dementia, also known as "childhood Alzheimer's." Mucopolysaccharidosis III can be classified into four subgroups (A, B, C, or D). Each subgroup is caused by a change in a particular enzyme (Table 2). Each subgroup is caused by a different enzyme deficiency and subsequently classified according to the specific deficient enzyme. Although each subtype of MPS III has its own specific enzyme deficiency, they all share similar clinical features that include cognitive decline and mental impairment, however, the severity of progression varies according to the type of MPS II, with MPS IIIA being the most severe [3]. Type A is most commonly reported in Northwestern Europe, type B in Southeastern Europe, and C and D types are less frequently reported [3]. The mode of inheritance of Sanfilippo syndrome is autosomal recessive. This pattern of inheritance has a higher prevalence (1:50,000 and 1:250,000) within consanguineous families [4].

**Table 2:** Mucopolysaccharidosis III subgroups.

Type	Gene	Enzyme
MPS IIIA	SGSH	N-sulphoglucosamine sulphohydrolase
MPS IIIB	NAGLU	α-N-acetylglucosaminidase
MPS IIIC	HGSNAT	Heparan-alpha-glucosaminide N-acetyltransferase
MPS IIID	GNS	N-acetylglucosamine-6-sulfatase

Classically, if both parents have a nonfunctional copy of a gene linked to this condition, their children have a 25% (1 in 4) chance of developing the disease [4]. Symptoms of the disease are likely to present early in life, particularly in the first year, and it can cause a decline in functional abilities. However, height may eventually fall below the average of their normal classmates, resulting in short stature. Among the signs and symptoms are delayed speech, behavior problems, specific features of autism spectrum disorder (difficulty with communication and social skills), sleep disturbances, developmental regression, intellectual disability, seizures, movement disorders, subtle coarse facial features, enlarged head (macrocephaly), enlarged tongue (macroglossia), umbilical hernia or inguinal hernia; children's symptoms may include arthritis, hearing loss, vision impairment, enlargement of the liver and spleen (hepatosplenomegaly), and frequent respiratory infections over time [5]. A variety of inborn errors of metabolism (IEM), including mucopolysaccharidosis III, were discovered in a previous prevalence study conducted in the Eastern province of Saudi Arabia. Glycogen storage, lysosome storage, mitochondrial, and other IEM abnormalities were described in Saudi Aramco Medical Services Organization facilities from a total population of 165,530 live births. There were three cases of MPS III recognized, two affected families, and an incidence rate of 2 per 100,000 live births [6]. A global incidence rate has been summarized in Table 3.

**Table 3:** Summary of reported prevalence studies of MPS III.

Country	Incidence (per 100,000 live births)			
	MPS IIIA	MPS IIIB	MPS IIIC	MPS IIID
Australia	0.78	0.43	0.07	0.09
France	0.46	0.10	0.09	0.03
Germany	1.11	0.36	0.10	0.00
Greece	0.00	0.78	0.15	0.00
The Netherlands	1.16	0.42	0.21	0.10
Northern Portugal	0.00	0.72	0.12	0.00
Sweden	0.44	0.03	0.17	0.00
Taiwan	0.08	0.28	0.03	0.00
United Kingdom	0.82	0.21	0.06	0.02

## Review

Methodology

Review Protocol

A review of the literature on medications and drugs used to treat Sanfilippo syndrome patients was conducted by three researchers who independently and blindly searched the following electronic databases: PubMed/MEDLINE and Embase using the following keywords: "Sanfilippo syndrome", "Mucopolysaccharidosis type III", and "MPS III". Interventional and observational clinical trials at all phases, which analyzed the outcome of different treatment methods to treat patients with Sanfilippo syndrome, were included using the US National Library of Medicine's database where 40 studies were identified, and those who met the inclusion criteria were thoroughly examined.

Inclusion Criteria

Included studies were selected according to the following criteria: Peer-reviewed articles/papers, articles that are written in English, clinical trials with a population aged 18 years and younger, articles indexed in PubMed/MEDLINE, Embase, and clinical trials indexed in the U.S. National Library of Medicine last updated on 2015/1/1.

Exclusion Criteria

The exclusion criteria were structured to ensure that the review focuses on relevant, up-to-date, peer-reviewed studies and clinical trials primarily targeting patients diagnosed with Sanfilippo syndrome, while excluding non-English materials, irrelevant studies, outdated trials, and duplications. Studies that do not meet our inclusion criteria, even after thorough examination, will be excluded. In case multiple articles or trials report on the same study, only the most comprehensive and recent publication will be included, while duplicates will be excluded. Furthermore, studies conducted solely on animal models without clinical relevance to human patients will be excluded.

Subject Population

The studies and clinical trials in this review targeted patients who were 18 years old and younger, encompassing both males and females. This age range reflects the focus on clinical trials mainly targeting pediatric patients with Sanfilippo syndrome, as described in the inclusion criteria.

Treatment and intervention

There is currently no approved curative medication for Sanfilippo syndrome that can effectively halt or reverse the disorder. Supportive therapies are indicated for visual disorders, hearing issues, neurodevelopmental disorders, cardiac involvement, seizures, and musculoskeletal complications [7]. As a result, the need for further research to develop an affordable and efficient treatment plan as well as recommending high-efficiency procedures remains an active area of research in medical practice, and developing one can aid in cost reduction and a better social environment for families with an affected patient. Recent research has primarily focused on expanding studies in promising treatment approaches such as using gene therapy to directly target the gene defect, using modified enzyme replacement to replace a defective or absent enzyme, using substrate reduction therapy (SRT) to administer an inhibitory molecule, using pharmacological chaperon to target incorrectly folded proteins and correctly fold the proteins and using stem cells to target the deficiency of the protein that the gene codes for. Although animal models have shown promise in terms of safety and clinical efficacy, clinical trials have proven to be costly and have yielded modest therapeutic outcomes [8].

Enzyme Replacement Therapy

Enzyme replacement therapy (ERT) is a medical technique that involves injecting the appropriate enzyme into the body to replace a damaged or absent enzyme. Some lysosomal storage diseases, such as Gaucher disease, Fabry disease, MPS I, MPS II (Hunter syndrome), MPS VI, and Pompe disease, are currently treatable with enzyme replacement treatment [9]. Endocytosis of lysosomal enzymes to the lysosome can occur via M6P receptors in the cell membrane [10,11]. Exogenous administration of the proper version of the mutant enzyme, such as sulfamide, NAGLU, or GNS, has laid the framework for non-neurological lysosomal storage disease therapy by being secreted from and absorbed by lysosomes within cells [11]. Until now, ERT has been the most promising technique [12]. However, for central nervous system (CNS) diseases, the blood-brain barrier (BBB), which decreases enzyme availability in the brain, has been shown to be a considerable impediment. Antibodies against the enzyme were also found in lysosomal storage disease patients who were treated, reducing the efficiency of the ERT [13]. This has been hampered in MPS IIIB by low absorption caused by insufficient mannose-6-phosphorylation (M6P) of the human α-N-acetylglucosaminidase recombinant enzyme. To address this issue, Kan et al. tested the use of a modified human recombinant NAGLU enzyme by fusing the human NAGLU fragment (rhNAGLU) to an insulin-like growth factor II fragment, which would allow trafficking into the lysosome, which was successful, and the enzyme remained functional and reduced the amount of Heparan Sulphate (HS) in MPS IIIB fibroblasts [14,15]. A phase IIb randomized open-label clinical trial on 21 MPS IIIA patients aged 12 to 48 months was conducted in 2018. They were randomly randomized (1:1:1) to receive no therapy (control), rhHNS IT 45 mg delivered once every two weeks (Q2W), or rhHNS IT 45 mg delivered once every four weeks (Q4W) for 48 weeks through an intrathecal drug delivery device placed in their chest. In three treated patients, a clinical improvement was noted (two in the Q2W group, one in the Q4W group). Heparan sulfate (HS) levels in cerebral fluid and urine GAG were decreased in all of the individuals. However, most secondary efficacy measures were nearly the same across treated patients (n=14; age, 17.8-47.8 months) and untreated controls (n=7; age, 12.6-45.0 months). Even though the major neurocognitive objective was not met, the levels of HS and GAG were lowered [16]. Intravenous ERT is now offered for MPS variants with minimal neurological impact. Recent MPS IIIA and MPS IIIB research has focused on modifying this traditionally systemic technique of accessing the CNS. Hijacking proteins, which easily cross the BBB, is one method. One approach is to use a molecular Trojan horse, which resulted in reduced GAG levels (72-83%) in Rhesus monkeys after intravenous administration of this fusion protein [17].

Substrate Reduction Therapy

**Figure 1:**
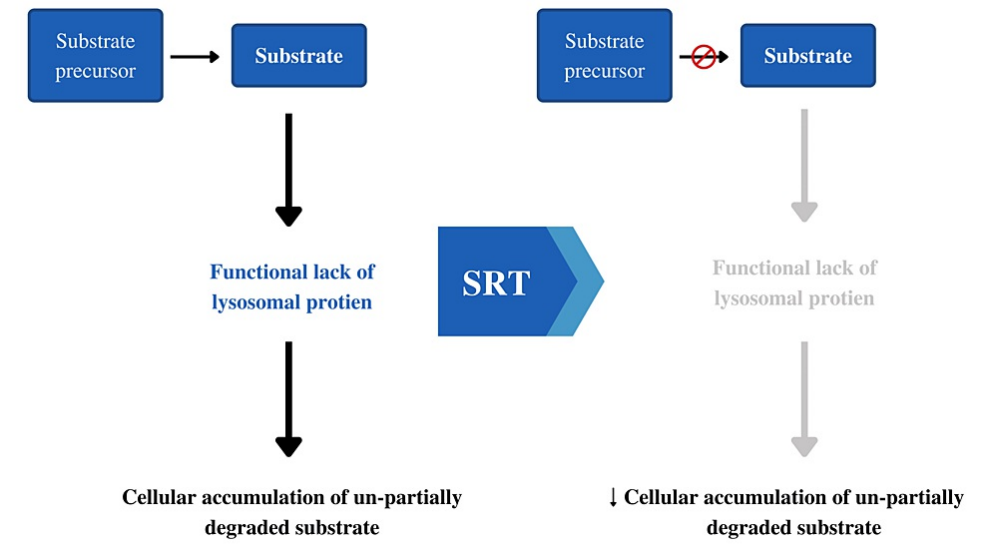
Substrate reduction therapy for lysosomal storage disorders. Image credit: Anas S. Alyazidi

SRT is a treatment technique in which an inhibitory molecule of glycolipid production is administered to restrict the substrate rate [18]. It is utilized to effectively clean the defective enzyme in individuals with glycolipid storage disorders [18,19]. SRT tries to restrict GAG synthesis in MPS III, which cannot be broken down in MPS III. It is critical to restore the balance of production and breakdown, and the mutant enzyme must retain some residual functions [18,19]. SRT has already been licensed for the treatment of a variety of lysosomal storage diseases, including those with neurological and non-neurological symptoms [18,19]. SRT is not permitted to treat any of the MPS disorders, however, other chemicals capable of crossing the BBB are being investigated for CNS therapy. SRT research is currently mostly focused on two ways [20]. The first is the use of genistein, a natural isoflavone extracted from soy that has been proven to reduce GAG levels by blocking follicle-stimulating hormone or epidermal growth factor, both of which have previously been found to enhance GAG production [20,21]. Although the precise mechanism by which this occurs is unknown, its capabilities as a kinase inhibitor enhance the activity of the transcription factor EB (TFEB). For nine months in 2006, researchers continually supplied genistein to MPS IIIB-affected mice [21]. This resulted in a decrease in GAGs and the restoration of behavioral abnormalities, synaptic loss, and clinical symptoms, which was made possible by its capacity to freely traverse the BBB [22]. Following these encouraging findings, a slew of human trials were launched. One of these was a phase III clinical research that was conducted in 2013; it was a double-blind study that employed a high dose of genistein against a placebo for 12 months [23]. Neuropsychological tests were performed on the patients before and after the study, and the results revealed that some patients improved while others stayed unchanged. The findings revealed that, while genistein reduced HS buildup, it had no effect on cognitive symptoms [24]. However, in a pilot research involving five MPS IIIA and five MPS IIIB patients, a genistein-enriched soy isoflavone extract was administered for 12 months, which lowered urinary GAG levels, improved hair morphology, and improved behavior [24]. Given the inconsistent results, more research with greater dosages of genistein and other flavonoids is needed to determine this set of chemicals' ability to alleviate CNS disease in Sanfilippo patients. Preclinical models (human and non-human studies) and clinical investigations of people with Sanfilippo syndrome reveal promising results for SRT. According to a recent evaluation, there is currently a study in phase III trial; it is the most promising trial so far (Figure 1) [25,26].

Pharmacological Chaperones for Enzyme-Enhancement-Therapy

**Figure 2:**
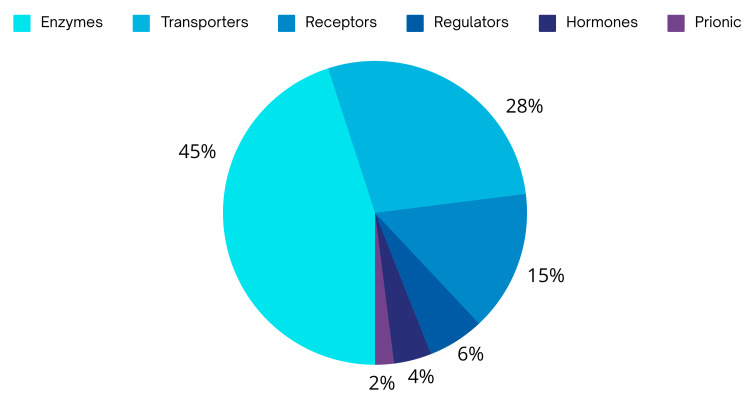
Protein targets of pharmacological chaperones based on their function. Image credit: Anas S. Alyazidi

A pharmacological chaperone, also known as a pharmacophore, is a new method in medicine that uses small-molecule ligands to target improperly folded proteins. They are a spatial arrangement of atoms or functional groups that represent the essential features of a molecule necessary for it to interact with a specific target receptor or enzyme and exert a pharmacological effect [27]. A pharmacological chaperone's protein targets can be various. Enzymes, particularly transferases, are the most numerous group (45%), followed by transporters (28%) and receptors (15%) (Figure 2) [28]. Many disorders, including Sanfilippo syndrome, are caused by mutations in certain genes. Protein mutations frequently result in molecular misfolding. Pharmacoperone acts to repair misfolded proteins' folding. It counteracts the effect of missense mutations by aiding proteins in folding correctly. Some enzymatic inhibitors, such as amino and iminosugars, may assist in the restoration of partial enzyme activity at low concentrations, which may be sufficient to avoid the associated symptoms [28]. Glucosamine, a competitive inhibitor of the HGSNAT enzyme, has shown promise in recent trials targeted at using chaperones as a therapy for MPS III [29,30].

The outcomes of the study demonstrated an increase in enzymatic activity in the fibroblasts studied, although more research is needed to address any potential shortcomings [30]. Future research in the field of pharmacological chaperone therapy will focus on the discovery of novel chaperones, including new allosteric medicines, as well as the utilization of synergies between chaperone treatment and other therapeutic techniques [28]. Pharmacological chaperones, which are currently being studied, are tiny molecule pharmaceuticals that can be chemically changed to be taken orally and cross the BBB. Although pharmacoperones might address the root cause of Sanfilippo syndrome, they are highly selective and may only be applied to some patients with Sanfilippo syndrome type B (NAGLU gene). Migalastat, an example of a successfully developed pharmacological chaperone, is currently approved in Australia for the treatment of another lysosomal storage disorder known as Fabry disease [31].

Stem Cell Therapy (SCT)

Stem cells are unspecialized cells that exist in both the embryo and the adult human organism. They have the ability to differentiate into certain cell types. They also have the ability to self-renew and differentiate into specialized adult cells. Adult stem cells (ASCs), embryonic stem cells (ESCs), and induced pluripotent stem cells (iPSCs) are the three types of stem cells. Hematopoietic stem cells (HSCs), mesenchymal stem cells, neural stem cells, epithelial stem cells, and skin stem cells are the different types of adult stem cells (Table 4) [32]. To avoid rejection, HSCs are produced from healthy donors' bone marrow or peripheral blood and implanted in patients after immunosuppression [32]. Stem cell treatment is a type of regenerative medicine that enhances the disease's healing response. HSCs are used to treat a number of metabolic diseases, including Hurler syndrome. Despite this, it has not been able to alleviate the symptoms of MPS III patients [33,34]. One trial on MPS patients found that, while HSCs effectively cured MPS in general, they were equivocal in MPS III individuals specifically [35]. An interventional study being done by an investigator at the Masonic Cancer Center at the University of Minnesota is one of the current clinical trials. Three volunteers under the age of 18 were given hematopoietic stem cell transplants. According to the stated neurologic outcomes, none of the three individuals participating in the study were evaluable for this endpoint. Two recipients received a second transplant, and one was lost to follow-up. This research was last updated on December 5, 2017 [36]. Another recent and larger study is presently underway at Duke University Medical Center, with the most recent update on October 8, 2020. The study includes 30 male and female participants who are being treated by hematopoietic stem cell infusion from living donors. The study is currently in its I/II phase, and its estimated primary completion date is April 2025, and the estimated completion date of the study is April 2028 [37].

**Table 4:** Stem cell therapy classification.

Classification	Type of cells
Adult stem cells	Hematopoietic stem cells
	Mesenchymal stem cells
	Neural stem cells
	Epithelial stem cells
	Skin stem cells
Embryonic stem cells	
Induced pluripotent stem cells	

Gene Therapy

Gene therapy is a medical technique that uses genes to treat or prevent disease. It is part of the ERT that primarily works by delivering a functional version of the gene into the cells to produce the essential protein or enzyme; gene therapy targets increasing or restoring defective enzyme activity in their cells and tissues [12]. The working gene needs to be delivered through a vector, usually a virus. Depending on the type of MPS and the tissue most affected by the disease, vectors may be delivered into the nervous tissue including the brain and the spinal cord; vectors can also be delivered into the liver or directly into the bloodstream. Gene therapy is intended to be a one-time procedure [38]. The gene can be delivered directly through a vector (i.e., in vivo gene therapy); other treatments being developed modify the cells outside the body (i.e., ex vivo gene therapy). In an open-label non-randomized trial, adeno-associated virus serotype 9 carrying the human NAGLU gene influenced by cytomegalovirus (CMV) enhancer/promoter (rAAV9.CMV.hNAGLU) was given intravenously one-time through a venous catheter. Targeted to treat Sanfilippo syndrome type B, the target sample included 15 patients. Abeona Therapeutics planned to finish the study by October 2022 [36,39]. In clinical trials conducted to address Sanfilippo syndrome type A, treatments included both biological and genetic intervention as well as drugs. One completed study in June 2017 conducted at Hôpitaux Universitaires Paris Sud, France by Dr. Kumaran DEIVA enrolled an actual number of four participants. The clinical study's main goal was to measure the long-term tolerability and safety of intracerebral administration of SAF-301 [40]. Although patients completed phase I/II of the study, information on neurological and cognitive changes, changes in potential biomarkers of the disease and further evaluation of immune response was set for a time frame of five years after the injection of SAF-301. Another study conducted at Manchester University NHS Foundation Trust will be the first in a human clinical trial to explore the safety, tolerability and clinical efficacy of ex vivo gene therapy (autologous CD34+ cells transduced with a lentiviral vector containing the human SGSH gene) in patients with MPS III type A. Following treatment with gene therapy patients will be followed up for a minimum of three years. The study has included an estimated number of five participants and is set to be completed on October 30, 2024 [41].

## Conclusions

In conclusion, the treatment of Sanfilippo syndrome is an active and evolving field of research. While curative medications are still under development, current therapeutic approaches are focused on targeting the underlying pathophysiology of the disease, such as refolding misfolded proteins. Ongoing clinical trials are providing promising results, and it is anticipated that some of these trials will be completed in the coming years. The treatment methods discussed in this review show potential in addressing Sanfilippo syndrome, but further investigations are necessary, particularly in larger samples. Each treatment approach holds significance and should be thoroughly researched and investigated. Gene therapy, ERTs, SRTs, pharmacological chaperones, and stem cell therapy are being explored as potential treatment options. Gene therapy has shown promise in early clinical trials for Sanfilippo types A and B. However, more research is needed to assess long-term safety and efficacy. ERT holds promise, but its effectiveness is limited by the BBB for neurological symptoms. Furthermore, pharmacological chaperones offer a potential approach to address the underlying cause of Sanfilippo syndrome in some patients. More research is required to identify new chaperones and explore their synergy with other therapies. Stem cell therapy using HSCs has shown limited success in patients with MPS III. Further clinical trials are ongoing to assess its efficacy and safety. While some of these approaches have shown promise in early studies, more research is needed to assess their long-term safety and efficacy. Continued research in this field is essential to address the challenges associated with this rare disease and ultimately improve the lives of affected individuals and their families.
